# CaMPARI2 enables stimulus-locked whole-brain activity mapping at cellular resolution in unrestrained larval zebrafish

**DOI:** 10.3389/fnmol.2026.1772915

**Published:** 2026-04-14

**Authors:** Kate R. Robbins, Amelia Bredbenner, Rebecca A. Osbaldeston, Kevin S. Villafañe, Eva E. Shin, Elizaveta Merkulova, Ava Clevenger, Payton B. Delean, Cristina Campos, Graham C. Peet, Roshan A. Jain

**Affiliations:** 1 Bi-College Interdisciplinary Neuroscience Program, Haverford College, Haverford, PA, United States; 2 Broad Institute of MIT and Harvard, Cambridge, MA, United States; 3 Department of Biology, Haverford College, Haverford, PA, United States; 4 Department of Medicine, Perelman School of Medicine, University of Pennsylvania, Philadelphia, PA, United States; 5 Department of Microbiology, Perelman School of Medicine, University of Pennsylvania, Philadelphia, PA, United States; 6 Department of Pediatrics, Lurie Center for Autism, Massachusetts General Hospital, Boston, MA, United States; 7 Bi-College Interdisciplinary Neuroscience Program, Bryn Mawr College, Bryn Mawr, PA, United States; 8 Neuroscience Program, Department of Cell and Developmental Biology, University of Colorado Anschutz Medical Campus, Aurora, CO, United States

**Keywords:** behavior selection, CaMPARI, escape response, habituation, learning, MK-801, zebrafish

## Abstract

Visualizing active neurons and circuits *in vivo* is critical for investigating the neural activity that underlies behavior. While several established methodologies are available to achieve this end in larval zebrafish, they are limited by the scale of tissue visualization, temporal resolution, need to restrain larvae, and/or accessibility of necessary instruments. Here, we establish a pipeline for the visualization and quantification of spatiotemporally precise whole-brain neural activity in larval zebrafish using CaMPARI2, a genetically-encoded calcium indicator. Using temporally specific photoconverting UV light exposures, we capture whole-brain “snapshots” of neural activity time-locked to stimuli during unrestrained larval behavior. We optimize experimental conditions for recording sub-second neuronal activity changes across acoustically-evoked behavioral paradigms spanning minutes to hours. We then leverage this system to pinpoint brain-wide neural activity changes during non-associative habituation learning, observing distinct activity signatures in the subpallium, preoptic area, and habenulae that are altered through pharmacological disruption of habituation learning. This approach effectively complements the temporal precision achievable through *post-hoc* activity detection methods and expands the accessibility of large-scale behavioral circuit dissection beyond highly specialized real-time volumetric imaging equipment.

## 1 Introduction

Visualizing large-scale neural activity is vital for understanding how neural circuits drive and modulate behavior. In vertebrates, several established methodologies enable neuronal activity monitoring. Fluorescent activity sensors, such as genetically-encoded calcium indicators (GECIs), neurotransmitter sensors, and voltage indicators, harness the transient intracellular calcium fluctuations and membrane voltage changes associated with neural action and communication (Miyazawa et al., 2018; Sakamoto and Yokoyama, 2025). Through reversible Ca^2+^-dependent fluorescence changes, GECIs such as GCaMP and its optimized and tailored variants enable live, sensitive monitoring of activity in defined neural circuitry with high temporal resolution (Chen et al., 2013; Zhang et al., 2023). While useful for investigating cellular and subcellular calcium dynamics, reversible indicators require real-time, continuous visualization during behaviors of interest because Ca^2+^- and voltage-dependent signals are transient (Abdelfattah et al., 2019; Hiyoshi et al., 2021; Kawashima et al., 2025). High-resolution imaging in small transparent vertebrates like zebrafish can effectively capture whole-brain dynamics at cellular resolution, where constraints on the visualizable tissue volume in larger organisms make it difficult to observe coordinated activity across non-adjacent neural populations (Scott et al., 2018; Vanwalleghem et al., 2018; Findling et al., 2025). These approaches typically require animal restraint or invasive implanted optical devices that may alter behavioral performance or limit how complex and generalizable recorded behaviors can be (Ahrens et al., 2012; Malvaut et al., 2020).

*Post-hoc* detection of endogenous neuronal activity markers provides an alternative strategy that enables whole-brain activity visualization without continuous imaging. Neuronal depolarization activates the Ras-ERK signaling pathway, leading to transcription factor phosphorylation and subsequent expression of immediate early genes (IEGs) such as *Arc* and *c-fos* (Xia et al., 1996; Herdegen and Leah, 1998; Thomas and Huganir, 2004; Guzowski et al., 2005). Detection of phosphorylated ERK (pERK) or IEG expression after animals undergo a behavioral task offers a readout of recent neural activity without needing to restrain animals or limit the scale of visualizable tissue (Barykina et al., 2022; Shainer et al., 2023). Despite these advantages, activity-dependent changes captured by pERK and IEG-based approaches occur on a range of minutes to hours, limiting temporal resolution (Randlett et al., 2015; Sauvage et al., 2019; Barykina et al., 2022). Additionally, suboptimal sensitivity, weak correlation between *Arc* and *c-fos* expression and neural activity, and the differential capacity of IEGs to detect activity across neural populations complicate interpretation of which neurons were active during a given period (Fields et al., 1997; Kovács, 2008; Chiaruttini et al., 2025). Critically, these methods report a summation of all neural activity occurring within their extended detection windows, obscuring circuit activity patterns associated with distinct, temporally precise responses, behaviors, or processing stages. Although whole-brain functional calcium imaging in freely behaving animals is possible, such systems are highly specialized and broadly inaccessible due to cost and complexity (Kim et al., 2017; Scott et al., 2018; Hasani et al., 2023). Thus, accessible approaches that capture temporally specific neural activity across large tissue volumes in freely behaving animals are needed to investigate how neural circuits modulate behavior.

CaMPARI is a photoconvertible GECI that enables whole-brain capture of neural activity at cellular resolution, which can be temporally refined through precise application of photoconverting light (Fosque et al., 2015). When elevated intracellular Ca^2+^ coincides with ultraviolet (UV) light, CaMPARI undergoes irreversible green-to-red photoconversion (PC), permanently marking neurons that were active during UV exposure to yield a whole-brain “snapshot” of temporally precise activity at cellular resolution (Fosque et al., 2015; Moeyaert et al., 2018; Ebner et al., 2019). Because the ratio of red to green fluorescence scales with intracellular calcium levels, CaMPARI and its enhanced variant CaMPARI2 enable robust quantification of differential neuronal activity across large tissue volumes in diverse organisms (Fosque et al., 2015; Moeyaert et al., 2018; Edwards et al., 2020; Das et al., 2023b). Pan-neuronal CaMPARI expression in zebrafish larvae has been leveraged to detect broad total brain activity differences in fish exposed to neurotoxins and drugs (Kanyo et al., 2021, 2023; Biechele-Speziale et al., 2023). However, there is untapped potential for using brain-wide CaMPARI2 in zebrafish to characterize patterns of neural activity with finer resolution associated with temporally fixed behavioral paradigms.

Acoustically-evoked startle behavior, being both temporally stereotyped and critically modulated, is well-suited to dissect with CaMPARI2 to understand the brain-wide neural activity patterns associated with non-associative learning in larval zebrafish. Startle behaviors like the rapid “C-start” escape responses are evoked by potentially threatening stimuli, and modulating when and how to respond to these stimuli is critical for survival across species (Koch, 1999; Evans et al., 2019; Zheng and Schmid, 2023). The well-characterized acoustically-evoked Short-Latency C-start (SLC) is driven by a pair of command-like rhombencephalic Mauthner cells that receive direct input from the statoacoustic ganglion, synapse onto contralateral spinal motor neurons, and are modulated by excitatory spiral fiber neurons as well as feed-forward and feed-back inhibitory glycinergic neurons (Burgess and Granato, 2007b; Koyama et al., 2011; Marsden and Granato, 2015; Hale et al., 2016). Repeated stimulus presentation induces robust non-associative habituation learning, characterized by a progressive reduction in SLC initiation probability (Rankin et al., 2009; Wolman et al., 2011). This fundamental form of learning enables organisms to adapt their responses to repetitive, non-threatening stimuli, promoting behavioral flexibility (Ardiel et al., 2017; Turatto et al., 2018; McDiarmid et al., 2019). Habituation differences are a well-documented feature of diverse human neuropsychiatric conditions including autism, attention-deficit hyperactivity disorder, schizophrenia, and anxiety (Jansiewicz et al., 2004; Meincke et al., 2004; Massa and O’Desky, 2012; Sladky et al., 2012; Avery and Blackford, 2016; Jamal et al., 2021; Dwyer et al., 2023). Involvement in etiologically diverse conditions suggests a role in context-dependent behavioral modulation and learning more broadly that whole-brain approaches may help elucidate (Lovett-Barron, 2021).

Here, we establish a pipeline for visualizing and quantifying spatiotemporally precise whole-brain neural activity in freely behaving larval zebrafish using CaMPARI2. Using acoustically-evoked escape behavior as a model, we validate that CaMPARI2 captures increased activity in neural populations previously implicated in acoustic perception, processing, and/or execution of the escape response. We demonstrate key experimental parameters allowing CaMPARI2 to capture and highlight temporally restricted events within long experimental paradigms. We then leverage this approach to pinpoint differences in neural activity associated with different habituation states in the subpallium, preoptic region, and habenulae, which are disrupted by pharmacological blockade of NMDA-type glutamate receptors that alters larval habituation state. Together, our findings position CaMPARI2 as an effective and accessible approach for resolving whole-brain, stimulus-locked, temporally-specific neural activity governing behavior in unrestrained larval zebrafish.

## 2 Results

### 2.1 CaMPARI2 captures acoustically-evoked activity through UV-dependent photoconversion

**FIGURE 1:**
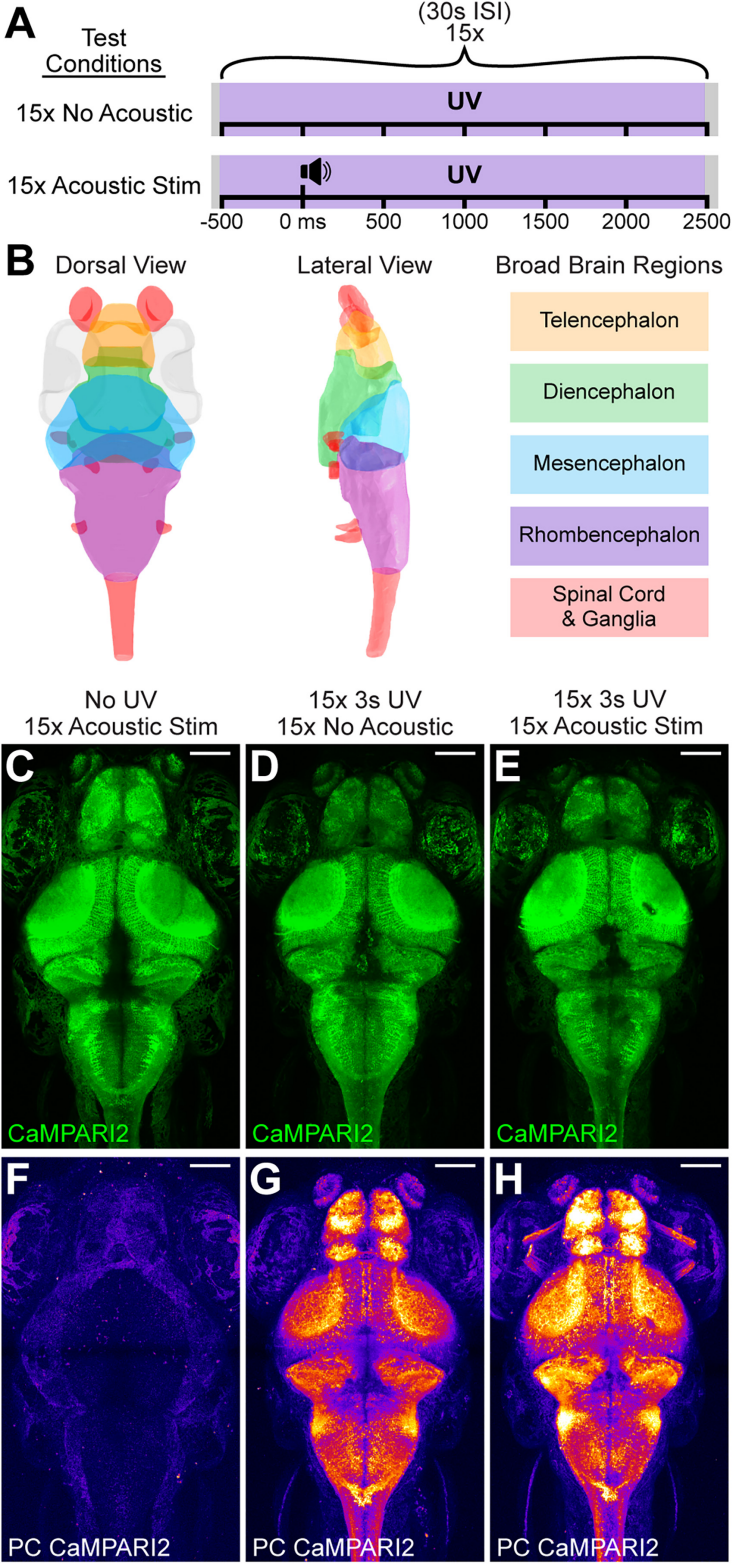
CaMPARI2 photoconversion is UV- and activity-dependent. **(A)** Schematic of UV and acoustic exposure conditions consisting of 15 UV exposures (3 s each), with or without a 2 ms acoustic stimulus (23.5 dB) presented 500 ms into each UV exposure. **(B)** Schematic of major regions of the 6 dpf larval brain with the color code used to annotate the regional locations of specific neurons or ROIs in all subsequent figures. Eyes are marked in gray. **(C–H)** Representative maximum intensity z-projections of the entire brains of individual 6 dpf PTU-treated larvae expressing *elavl3:CaMPARI2* that were exposed to 15 acoustic stimuli without UV exposure **(C,F)**, 15 UV exposure events (3 s each) without acoustic stimuli **(D,G)**, or 15 UV exposures each combined with an identical acoustic stimulus **(E,H)**. Unconverted CaMPARI2 is in green **(C–E)**, and photoconverted (PC CaMPARI2) red fluorescence is pseudocolored by intensity to visualize differences in photoconversion **(F–H)**. White scale bar represents 100 μm.

Since CaMPARI2 photoconversion requires UV light to capture neural activity, we first assessed whether UV-dependent photoconversion could be detected when CaMPARI2 was expressed pan-neuronally in larvae. We exposed 6 days post-fertilization (dpf) larvae free-swimming in 9 mm diameter wells to 15 flashes of UV light (3 s each), separated by 30 s interstimulus intervals (ISI) (Figure 1A), then anesthetized and imaged whole-brain volumes with a line-scanning confocal microscope (Figures 1B–H). Baseline photoconverted red signal was minimal in larvae without experimental UV exposure (Figures 1C,F), whereas larvae exposed to UV light showed green-to-red photoconversion (PC) throughout the brain, indicating widespread capture of neural activity (Figures 1D,G). To see if we could capture brain activity differences due to acoustic stimulation, we presented larvae with a similar UV exposure paradigm in which an intense 2 ms acoustic stimulus was delivered 500 ms after each UV onset, maintaining the same 45 s total UV exposure time across the experiment (Figure 1A). Larvae exposed to concurrent acoustic stimuli and UV light displayed higher levels of CaMPARI2 PC across many brain regions (Figure 1H) than larvae exposed to UV light alone (Figure 1G), indicating that CaMPARI2 can capture acoustically-evoked neural activity in free-swimming larvae.

**FIGURE 2:**
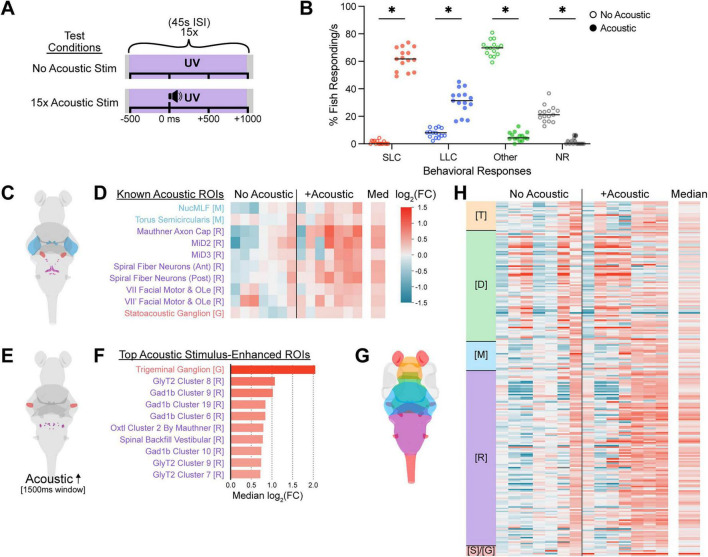
CaMPARI2 captures regional neuronal activity differences underlying acoustically-evoked behaviors. **(A)** Schematic of UV exposure conditions consisting of 15 UV exposures (1.5 s each), with or without 2 ms acoustic stimuli presented 500 ms into each UV exposure. **(B)** Percent of tracked 6 dpf wild type TLF larvae responding to 15 × 1.5 s UV at 45 s ISI with no acoustic stimuli (open circles, *n* = 57 larvae) or paired with acoustic stimuli (solid circles, *n* = 57 larvae). Behaviors tracked over 1 s following acoustic stimuli were classified into SLC escapes (red), LLC escapes (blue), routine turns and swims (Other, green), or non-responses (NR, gray). Each point represents the population-level percentage of fish responding with the given behavior for one stimulus, and black bars indicate the median percentage for each behavior. * Indicates significance by multiple Mann-Whitney tests with a 0.05 corrected *p*-value threshold, Holm-Šídák method. **(C–H)** Diagrams and heatmaps of log_2_(fold change) in neural activity of ROI in larvae in acoustically stimulated (*n* = 7 larvae) and unstimulated conditions (*n* = 7 larvae), relative to the median of the unstimulated condition, displaying a preselected subset of ROIs associated with acoustic perception, processing, or response **(C,D)**, the ROIs with the ten largest median activity increases in the acoustically stimulated condition relative to the unstimulated condition **(E,F)**, and across the entire brain **(G,H)**, with the corresponding color-coded ROIs diagrammed **(C,E,G)**. Each column presents data from separate individual fish imaged under unstimulated or acoustically stimulated conditions and each distinct row represents distinct specified ROI. The median log_2_(FC) values across all acoustically stimulated larvae is presented in the right-most column **(D,H)**. The log_2_(FC) legend in **(D)** corresponds to all bar colors **(D,F,H)**. ROIs are grouped and annotated by the major brain region in which they are found and colored as in Figure 1B: Telencephalon (T), Diencephalon (D), Mesencephalon (M), Rhombencephalon (R), and Spinal Cord and Ganglia (S)/(G). Within each condition, individuals were reordered from smallest to largest by the median log_2_(FC) value of each brain. ROIs with no detectable red or green fluorescence in an individual are shown in gray. Individual larvae and ROIs with > 10% missing red or green fluorescence measurements were excluded from analysis, leaving 243 ROIs in this data set.

Having validated UV- and stimulus-dependent photoconversion, we sought to increase the specificity of acoustically-evoked activity capture by scaling back the duration of each UV light exposure in our paradigm to 1.5 s (Figure 2A). While relatively stereotyped and well-characterized, acoustically-evoked escape behavior can still be significantly modulated by visual input (Mu et al., 2012; Marquart et al., 2019; Martorell and Medan, 2022; Otero-Coronel et al., 2024) and larvae of this age can detect UV through retinal cones (Guggiana-Nilo and Engert, 2016), so we assessed wild type larval escape behavior performance in the presence of photoconverting UV light (Figure 2B). Without intense acoustic stimuli, ∼20% of larvae remained stationary during 1 s of UV illumination (Figure 2B, NR), while most wild type larvae performed at least one movement bout during silent 1 s UV exposure windows (Figure 2B). Larvae rarely initiated high-speed SLC escape maneuvers or Mauthner-independent Long-Latency C-start (LLC) escape bouts without acoustic stimuli (Figure 2B), consistent with routine turn and swim bout initiation rates observed in other illuminated contexts (Burgess and Granato, 2007a; Groneberg et al., 2020; Zúñiga Mouret et al., 2024). In contrast, introducing a single acoustic stimulus during each UV illumination period drastically shifted the population-level behavioral profile, with nearly all wild type fish initiating a movement bout, most commonly an escape maneuver (Figure 2B, 61.7% SLC and 31.4% LLC median per 1 s window). We then captured the whole-brain neural activity patterns of 6–7 dpf *elavl3:CaMPARI2* larvae using these shortened UV paradigms (Figure 2A) providing 22.5 s total UV in the absence and presence of acoustic stimuli, and imaged whole-brain volumes with a line-scanning confocal microscope as before. UV-exposed larvae again displayed high levels of PC CaMPARI2 throughout the brain, so we compared larvae with and without acoustic stimulation at several z-sections, at manually matched brain depths based on structural landmarks (Supplementary Figure 1). Regional activity differences were visible between acoustically-stimulated and unstimulated larvae, both in individual neurons and neuropil areas (Supplementary Figure 1, see arrowheads and brackets), suggesting that large-scale alignment and comparison throughout the brain could reveal differential neuronal activity patterns underlying the different behavioral profiles of these populations.

To identify which neural populations were differentially activated by acoustic stimuli and enable comparison across experimental conditions and individuals, we adapted registration and quantification pipelines originally developed for whole-brain pERK and gene expression analyses in zebrafish larvae (Randlett et al., 2015; Shoenhard and Granato, 2023). Briefly, this involved registering each whole-brain confocal z-stack to a previously-established reference brain in the Z-Brain Atlas (Randlett et al., 2015), applying a Gaussian blur to improve regional signal consistency, quantifying green and red fluorescence across the Z-Brain atlas’ 293 spatially defined neuroanatomical regions of interest (ROIs), and then calculating a red-to-green ratio (RGR) for each ROI to correct for differential CaMPARI2 expression levels between ROIs and individuals. Since the neural activity captured by CaMPARI2 is expected to include both acoustically relevant activity and activity associated with other simultaneous neural processes, we calculated the fold change in fluorescence in larvae exposed to both acoustic stimuli and UV light relative to larvae exposed to UV light alone for each ROI. Based on our behavioral results, we predicted that neural populations specifically involved in initiation and execution of escape behaviors would show enhanced activity in acoustically-stimulated fish. Thus we selected 10 ROIs containing neurons previously implicated in perception, processing, and/or response to acoustic stimuli (Kohashi and Oda, 2008; Hale et al., 2016; Poulsen et al., 2021; Favre-Bulle et al., 2025), and assessed their fold increase in activity relative to the matched silent photoconversion cases [log_2_(FC)], (Figures 2C,D). We excluded the ROI containing the Mauthner cell bodies from our analyses due to an absence of CaMPARI2 expression in these cells in this transgenic line. While larvae exposed to acoustic stimuli displayed modest median activity increases in these acoustically-relevant ROIs, significant replicate-to-replicate variability was also evident (Figure 2D). Notably, the ROI containing the trigeminal ganglion, which directly evokes SLC escape behaviors in response to tactile stimuli, showed the greatest stimulus-dependent activity increase (Figures 2E,F; Kimmel et al., 1990; Yokogawa et al., 2012). Other ROIs exhibiting activity increases were primarily rhombencephalic and inhibitory, consistent with extensive activation of GABAergic and glycinergic hindbrain neurons during diverse swimming behaviors (Figures 2E,F; Severi et al., 2018). Across the entire brain, an overall increase in neural activity was captured in the acoustically-stimulated condition, though we also noted some within-group divergence in activity patterns (Figures 2G,H). This variability may reflect divergent behavioral profiles of the individuals in response to the 15 stimuli, variable UV-evoked behaviors, and/or spontaneous behavioral differences in the windows before or after acoustically-evoked escape bouts captured by UV.

### 2.2 Maximizing temporal restriction and specificity in acoustically-evoked activity capture

**FIGURE 3:**
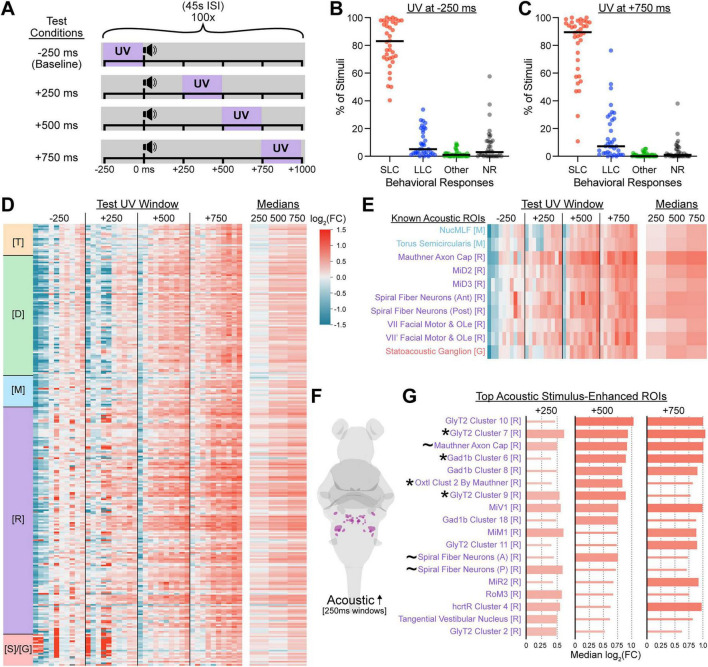
UV timing confers temporal restriction of neural activity captured by CaMPARI2. **(A)** Schematic of relative timings of acoustic stimuli and UV exposures. Each condition consisted of 100 intense acoustic stimuli presented at 45 s ISI and paired with 250-ms UV exposures beginning 250 ms before each stimulus onset (–250 ms, baseline), or 250, 500, or 750 ms after each stimulus onset (+250 ms, +500 ms, or +750 ms, respectively). **(B,C)** Frequencies of behavioral responses by 6 dpf wild type TLF larvae to acoustic stimuli delivered according to the paradigms in **(A)** where UV was presented 250 ms prior to acoustic stimuli (**B**, *n* = 36 larvae) or 750 ms following acoustic stimuli (**C**, *n* = 36 larvae). Larval behaviors were classified as in Figure 2B. Each point represents the percent of stimuli that an individual responded with the indicated behavior across all 100 stimuli, and black bars represent the median percentage for each behavior. **(D,E)** Heatmaps of log_2_(FC) in neural activity of ROIs of larvae in pre-stimulus (–250 ms, *n* = 10 larvae) and post-stimulus (+250 ms, +500 ms, +750 ms, *n* = 10 larvae each) 250-ms UV exposure conditions relative to the median of the pre-stimulus condition, across the entire brain **(D)** and the preselected “Known Acoustic ROIs” **(E)**. Columns represent individual larvae and rows represent individual ROIs (280 total included here). The rightmost columns show the median log_2_(FC) across all larvae in each post-stimulus condition. **(F,G)** ROIs from **(D)** displaying the ten largest median activity increases for each post-stimulus capture group relative to the pre-stimulus condition, with the corresponding color-coded ROIs diagrammed **(F)** and quantified **(G)**. Thicker bars indicate the ROI was among the top 10 median log_2_(FC) values for that condition. The log_2_(FC) legend **(D)** corresponds to all bar colors **(D,E,G)**. Individuals (columns) and ROIs (rows) are ordered, colored, and annotated by brain region as described in Figure 2. * Indicates the ROI was among the top 10 enhanced in Figure 2, and ∼ denotes ROIs from the “Known Acoustic ROI” subset.

A potential strength of the CaMPARI2 system relative to IEG-based methods is its ability to selectively capture discrete epochs of brain activity across an extended experimental time course. Because acoustically-evoked escape bouts initiate within 15 ms or 60 ms (for Mauthner-dependent SLC escapes or Mauthner-independent LLC escapes, respectively) and larval movement bouts typically last ≤ 200 ms, we restricted each UV photoconversion window to 250 ms (Burgess and Granato, 2007b; Marques et al., 2018). To maintain a similar total amount of UV exposure per fish relative to prior experiments, we exposed larvae to 100 acoustic-coupled UV exposures at 45 s ISI (Figure 3A). Larvae experienced 25 s of total UV exposure distributed over a 75-min experiment, capturing 100 behavioral epochs instead of 15. To explicitly test whether CaMPARI2 could selectively capture temporally-resolved acoustically-evoked neural activity, we assigned larvae to one of four different testing conditions, each receiving the same 100 identical acoustic stimuli varying only in the timing of 250-ms UV exposure relative to stimulus delivery (t = 0 ms): –250 ms, +250 ms, +500 ms, or +750 ms (Figure 3A). Because UV exposure in the –250 ms condition always concludes *before* the acoustic stimulus and subsequent escape initiation, we predicted that this condition would not capture acoustically-relevant activity. Importantly, we assessed larval behavior in response to these acoustic stimuli in the –250 ms and +750 ms experimental conditions, and found that both paradigms evoked very similar behavioral outcomes, with larvae performing mainly Mauthner-dependent SLC escapes and some Mauthner-independent LLC escapes (Figures 3B,C).

To identify consistent stimulus-locked activity, we calculated log_2_(FC) values relative to the median of the –250 ms condition for each ROI in each fish (Figure 3D). While whole-brain activity patterns of the –250 ms and +250 ms conditions displayed marked variance between replicates, within-group replicates of the +500 ms and +750 ms conditions were far more similar, with elevated activity consistently observed across individuals in many ROIs (Figure 3D). Focusing on our acoustic ROI set, fold change increased in magnitude from the +250 ms to the +750 ms condition (Figure 3E). We extracted the 10 ROIs with the highest fold change for each condition (Figure 3G, thick bars)—all were located in the hindbrain, and several of our pre-selected acoustic ROIs now appeared among the most differentially active ROIs for at least one condition (Figures 3F,G, ∼), alongside other descending reticulospinal clusters active during escape responses and vigorous swimming (MiV1, MiM1, MiR2, RoM3) (Gahtan et al., 2002; Lau et al., 2025). We observed substantial overlap in top-ranked ROIs between conditions such that most regions appeared among the top ROIs for multiple conditions (Figure 3G), consistent with a similar behavioral profile in each condition. However, the level of activity enrichment was always highest in the +750 condition, with 50–100% higher activity captured than even the top ROI for the +250 condition (Figure 3G). Taken together, introducing a 750-ms delay between acoustic stimulus delivery and UV light exposure confers sufficient temporal restriction to isolate acoustically-evoked escape behavior.

**FIGURE 4:**
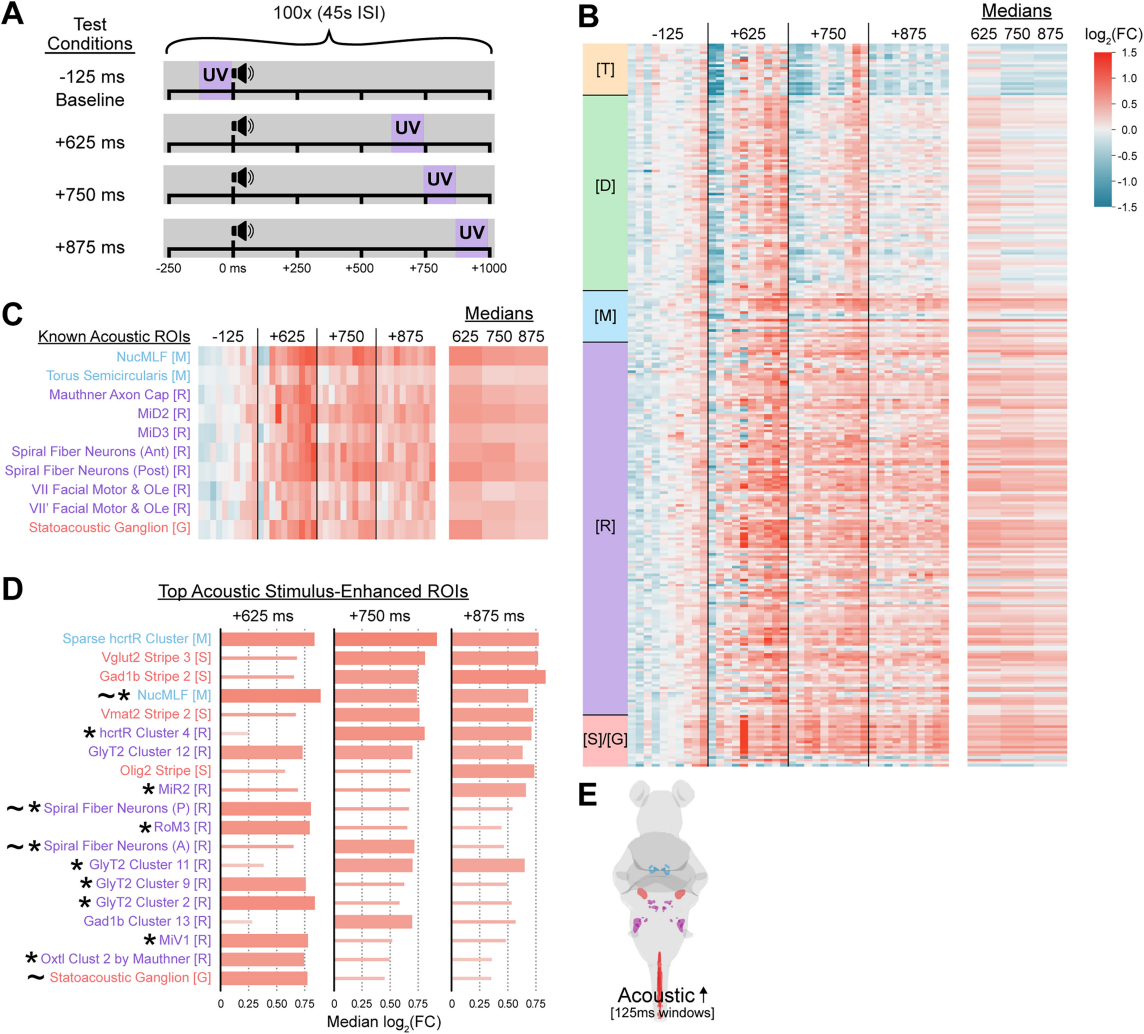
CaMPARI2 neural activity capture can be further refined through shortened photoconversion windows. **(A)** Schematic of relative timings of acoustic stimuli and UV exposures. Each condition consisted of 100 intense acoustic stimuli presented at 45 s ISI and paired with 125-ms UV exposures beginning 125 ms before each stimulus onset (–125 ms, baseline), or 625, 750, or 875 ms after each stimulus onset (+625 ms, +750 ms, or +875 ms, respectively). **(B,C)** Heatmaps of log_2_(FC) in neural activity of ROIs of larvae in pre-stimulus (−125 ms, *n* = 10 larvae) and post-stimulus (+625 ms, +750 ms, or + 875 ms, *n* = 10 larvae for each) 125-ms UV exposure timing conditions relative to the medians of the pre-stimulus condition, across the entire brain **(B)** and a selected subset of preselected acoustically-active ROIs **(C)**. Columns represent individual larvae and rows represent individual ROIs (281 total included here). The rightmost columns shows the median log_2_(FC) across all larvae in each post-stimulus condition. **(D,E)** ROIs displaying the ten largest median activity increases in post-stimulus conditions relative to the pre-stimulus condition, with the corresponding color-coded ROIs quantified **(D)** and diagrammed **(E)**. Thicker bars indicate the ROI was among the top 10 median log_2_(FC) values for that condition. The log_2_(FC) legend **(B)** corresponds to all bar colors **(B–D)**. Individuals (columns) and ROIs (rows) are ordered, colored, and annotated by brain region as described in Figure 2. * indicates the ROI was among the top ten enhanced in Figures 2, 3, and ∼ denotes ROIs from the “Known Acoustic ROI” subset.

We next assessed whether further temporal restriction of the UV capture period could still efficiently detect hindbrain activity linked to escape behaviors. We thus refined our UV exposure period to 100 bouts of 125 ms UV, timing the UV initiation relative to each stimulus as follows: –125 ms (pre-stimulus baseline), +625 ms, +750 ms, and +875 ms (all post stimulus) (Figure 4A). This more restricted UV window still reliably captured consistent and widespread elevated activity in many ROIs of the hindbrain relative to the baseline, though broad activity in the more rostral brain areas was more muted (diencephalon) or reduced (telencephalon) for the +750 and +875 ms conditions (Figure 4B). Acoustically relevant ROIs again indicated capture of escape-related brain activity with peak median fold change values generally found in the +625 ms condition (Figure 4C). Top ranked ROIs were relatively consistent between +750 and +875 ms conditions, with many consistent ROIs being detected that were also top ranked when longer UV windows were used (Figures 4D,E, asterisks), validating highly restricted capture of acoustically-evoked escape behavior.

### 2.3 CaMPARI2 captures differences in habituation learning states

**FIGURE 5:**
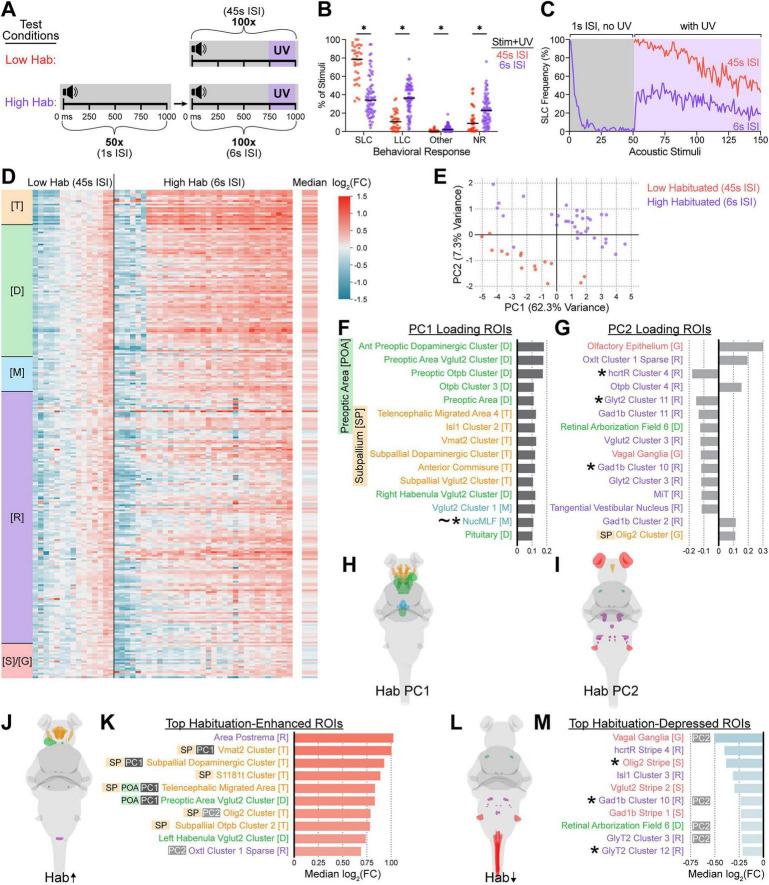
CaMPARI2 captures differential acoustically evoked neural activity between low- and high-habituating larvae. **(A)** Schematic of relative timings of acoustic stimuli and UV exposures. The low-habituating (Low Hab, top) condition consisted of 100 intense acoustic stimuli with a 45 s ISI and accompanying 250-ms UV exposure beginning 750 ms after each acoustic stimulus onset as in Figure 3A. The high-habituating (High Hab, bottom) condition consisted of 50 intense acoustic stimuli presented at 1 s ISI without accompanying UV exposure followed immediately by 100 intense acoustic stimuli presented at 6 s ISI with accompanying 250-ms UV exposures beginning 750 ms after each acoustic stimulus. **(B,C)** Frequencies of behavioral responses by 6 dpf wild type TLF larvae to the 100 UV-coupled acoustic stimuli delivered in the Low Hab (red, *n* = 36 larvae) or High Hab (violet, *n* = 71 larvae). Larval behaviors were classified as in Figure 2B. Each point represents one individual across all 100 UV-coupled stimuli **(B)**, with black bars representing the medians, * Indicates significance by multiple Mann-Whitney tests with a 0.05 corrected *p*-value threshold, Holm-Šídák method. Average frequencies of SLC responses across the entire experimental course are presented in **(C)**. **(D)** Heatmap of log_2_(FC) in neural activity of ROIs of individual larvae in low-habituating (45 s ISI, *n* = 15) and high-habituating (6 s ISI, *n* = 33) conditions, relative to the medians of the low-habituating condition. Columns represent individual larvae and rows represent individual ROIs (281 total included here). The median log_2_(FC) for all High Hab condition larvae relative to the Low Hab condition is presented in the right-most column. **(E)** Principal component analysis (PCA) of ROI-level RGRs from **(D)**. Each point represents an individual larva from the low-habituating condition (red) or the high-habituating condition (violet). X- and Y-axes show the first (PC1) and second (PC2) principal components, accounting for 62.3% and 7.3% of the total variance, respectively. **(F–I)** ROIs with the 15 largest loadings on PC1 **(F)** and PC2 **(G)**, respectively diagrammed in **(H,I)**. Bar lengths indicate loading value. **(J–M)** ROIs displaying the ten largest median activity increases **(J,K)** and decreases **(L,M)** in the high-habituating condition relative to the low-habituating condition, with the corresponding color-coded ROIs diagrammed **(J,L)** and quantified **(K,M)**. The log_2_(FC) legend **(D)** corresponds to bar colors **(D,K,M)**. Individuals (columns) and ROIs (rows) are ordered, colored, and annotated by brain region as described in Figure 2. * Indicates the ROI was among the top 10 enhanced in Figures 2–4, ∼ denotes ROIs from the “Known Acoustic ROI” subset. PC1 loading [(PC1), dark gray] and PC2 loading [(PC2), light gray] ROIs are marked, as are ROIs in or overlapping the preoptic area (POA) and subpallium (SP).

Having successfully established temporal restriction in CaMPARI2 activity capture through optimized UV light delivery conditions, we leveraged the temporal control of CaMPARI2 to reveal brain-wide activity differences underlying different behavioral states of habituation. With repeated acoustic stimuli, zebrafish first shift their behavioral bias from performing Mauthner-dependent SLC escapes toward responding with Mauthner-independent LLC escapes (Jain et al., 2018). Larvae eventually discontinue motor responses to the stimuli, following conserved features of habituation learning where shorter ISIs between repeated stimuli produce faster learning and stronger behavioral attenuation in fewer stimuli than longer ISIs (Rankin et al., 2009; Wolman et al., 2011; Jain et al., 2018). We thus adapted our stimulation paradigm to capture brain activity when fish experience acoustic stimuli in a higher habituation state by pre-exposing fish to an extra 50 stimuli at 1 s ISI, which were followed by 100 UV-coupled stimuli at 6 s ISI (Figure 5A). This captured acoustically-evoked brain activity where larvae are more likely to perform LLCs or not respond compared to matched fish receiving only 100 UV-coupled acoustic stimuli at 45 s ISI, where responses are primarily SLCs (Figure 5B). Importantly, these two stimulation paradigms received identical amounts of photoconverting UV light capturing an identical number of UV-accompanied stimuli (250 ms UV exposure initiated 750 ms after each of 100 stimuli). Thus the “Low Habituating” 45 s ISI condition captures the transition of naïve fish beginning to shift their bias away from SLCs, and the “High Habituating” 6 s ISI condition captures brain activity after fish have already shifted their behavioral profile (Figure 5C).

We next directly compared the captured brain activities between the Low and High Habituation conditions and observed distinct patterns of regional activity increases and decreases across high-habituating individuals relative to low-habituating individuals (Figure 5D). Notably, broad patterns of relative regional increases and decreases appeared dissimilar to those observed in post-stimulus vs. pre-stimulus conditions, with higher median activities in more rostral brain regions and less consistent activity increases in the rhombencephalon (Figure 5D). Principal component analysis (PCA) of whole-brain patterns of ROI-level activity segregated high- and low-habituating larval groups into two distinct clusters separating along both PC1 and PC2, accounting for 62.3% and 7.3% of variance, respectively (Figure 5E). This revealed robust and consistent multivariate activity differences between the high- and low-habituating conditions, establishing the two behavioral states as discrete (Figure 5E). Examining PC1 and PC2 loadings identified the ROIs contributing most to discrete activity patterns between groups (Figures 5F,G). PC1 was largely dominated by telencephalic and diencephalic ROIs, while PC2 was negatively loaded by many rhombencephalic ROIs, including several of the top ROIs from the previous low-habituating experiments (Figures 5F–I, asterisks). Several ROIs loading most strongly into PC1 were also among those most differentially active in the positive direction (Figure 5K). ROIs displaying the most significantly reduced activity in the high vs. low habituating condition also overlapped with ROIs previously identified as having increased activity in stimulated larvae relative to no-acoustic or pre-acoustic UV capture larvae (Figure 5M, asterisks).

**FIGURE 6:**
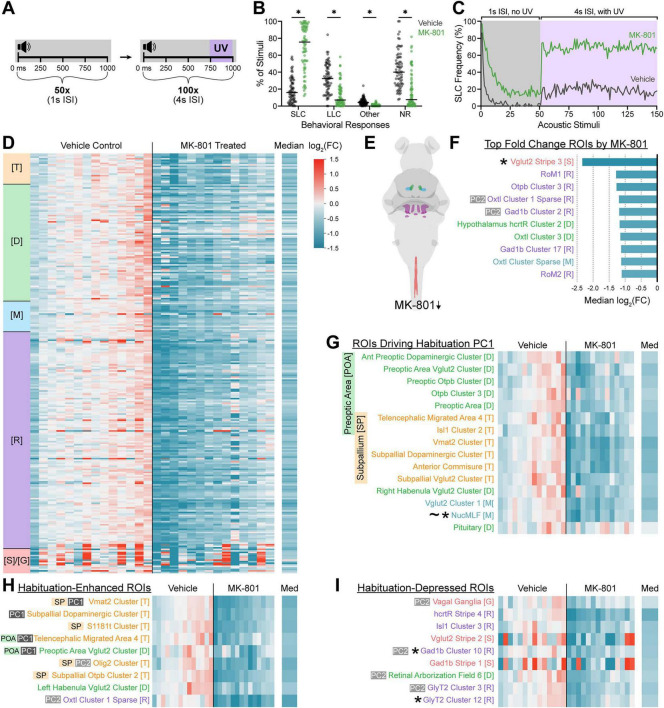
CaMPARI2 captures broad brain state differences when habituation is pharmacologically impaired by MK-801. **(A)** Schematic of relative timings of acoustic stimuli and UV exposures. Larvae pretreated for 30 min with vehicle or 500 μM MK-801 were subjected to 50 intense acoustic stimuli presented at 1 s ISI without accompanying UV exposure, followed immediately by 100 intense acoustic stimuli presented at 4 s ISI with accompanying 250-ms UV exposures beginning 750 ms after each acoustic stimulus. **(B,C)** Frequencies of behavioral responses by 6 dpf wild type TLF larvae to the 100 UV-coupled acoustic stimuli of the paradigm described in **(A)** following pretreatment with vehicle (gray, *n* = 72 larvae) or 500 μM MK-801 (green, *n* = 72 larvae). Larval behaviors were classified as in Figure 2B. Each point represents one individual across all UV-coupled stimuli **(B)**, black bars represent the median, and * indicates significance by multiple Mann-Whitney tests with a 0.05 corrected *p*-value threshold, Holm-Šídák method. Average frequencies of SLC responses across the entire experimental course are presented in **(C)**. **(D–I)** Heatmaps of log_2_(FC) in neural activity of ROIs of individual larvae in vehicle-treated (*n* = 14 larvae) and MK-801-treated conditions (*n* = 14 larvae), relative to the median vehicle-treated condition for each ROI. ROIs displaying the ten largest median activity decreases in the MK-801-treated condition relative to the vehicle-treated condition are diagrammed **(E)** and quantified **(F)**. Heatmaps of log_2_(FC) from the MK-801-treated and vehicle-treated larvae from **(D)** for notable ROI subsets identified in Figure 5 include: The 15 ROIs contributing most strongly into habituation PC1 **(G)**, the top ROIs enhanced in high habituation conditions of normal fish **(H)**, and the top ROIs depressed in high habituation conditions of normal fish **(I)**. Columns represent individual larvae and rows represent individual ROIs (273 total included here). The rightmost column shows the median log_2_(FC) across all MK-801-treated larvae. The log_2_(FC) legend **(D)** corresponds to bar colors **(D,F–I)**. Individuals (columns) and ROIs (rows) are ordered, colored, and annotated by brain region as described in Figure 2. * Indicates the ROI was among the top acoustically-enhanced in Figures 2–4, ∼ denotes ROIs from the “Known Acoustic ROI” subset. Habituation PC1 loading [(PC1), dark gray] and PC2 loading [(PC2), light gray] ROIs from Figure 5 are marked, as are ROIs overlapping or within the preoptic area (POA) and subpallium (SP).

To further explore the neural basis of the non-associative learning process, we pharmacologically disrupted acoustically-evoked habituation learning by pretreating larvae with NMDA-type glutamate receptor (NMDAR) blocker MK-801 or vehicle alone for 30 min, then captured brain activities for both groups of larvae with a high-habituation stimulus paradigm (Figure 6A). To induce more rapid and robust habituation, we shortened the habituating ISI from 6 s to 4 s. Similar to untreated larvae (Figure 5B), vehicle-treated larvae were pre-shifted to a more habituated state for the photoconversion phase with higher rates of non-response or LLC responses than SLC responses (Figure 6B, gray). In contrast, NMDAR-disrupted larvae largely responded to the same stimulus paradigm with Mauthner-dependent SLC responses and most individuals showed low frequencies of LLC responses or non-responses (Figure 6C, green), reflecting their predicted habituation learning deficit (Wolman et al., 2011; Marsden and Granato, 2015; Nelson et al., 2023; Köcher and Straumann, 2025). Comparing brain-wide activity patterns between vehicle and MK-801 treated larvae revealed widespread relative reductions in activity across all broad structural regions of the brain (Figure 6D), consistent with the global pharmacological disruption of excitatory glutamate neurotransmission. ROIs featuring the largest activity changes were largely rhombencephalic with reduced activity relative to vehicle-treated controls (Figure 6E), and these included multiple ROIs that positively loaded to Habituation PC2 (Figures 6E,F, PC2). We specifically examined the ROIs driving Habituation PC1 to look for disrupted activation by MK-801, and all but the pituitary showed median activity reductions (Figure 6G). Similarly, the top habituation-enhanced ROIs all showed significant median fold changes in activity (Figure 6H), consistent with the behavioral reduction in habituation by MK-801. We also observed somewhat milder reductions in the median fold change of the top habituation-depressed ROIs, though these notably showed high variance between individuals (Figure 6I). Together these data indicate that CaMPARI2 captures the neural activity changes associated with large-scale pharmacological circuit disruption.

## 3 Discussion

While visualizing neural activity is fundamental to understanding dynamic control of behavior, doing so with high temporal specificity in unrestrained animals proves challenging. Here we refine an approach to capturing temporally restricted, stimulus-locked neural activity in free-swimming larval zebrafish with CaMPARI2. By using the tool to investigate acoustically-evoked escape behavior, a temporally fixed paradigm initiated by spatially diverse neuroanatomical regions, we demonstrate its utility for elucidating differential activity patterns between larvae in various behavioral and learning states.

### 3.1 Optimizing key variables for temporally specific CaMPARI2 activity capture

Selecting the appropriate timing and intervals of photoconverting UV light is crucial for isolating behaviorally relevant neural activity from background signal. Comparing individuals receiving 15 UV exposures within-group in the absence or presence of acoustic stimuli revealed individual patterns with activity values diverging extensively from group medians, suggesting substantial capture of irrelevant spontaneous neural activity and/or biological behavioral state differences (Figure 2H). Consistent with this interpretation, acoustically relevant ROIs showed similarly variable activity patterns between replicates, with only modest and inconsistent activity increases in populations known to be critical for acoustically evoked escape (Figure 2D). Identifying such activity patterns that are relevant can be enhanced by reducing noise from individual-to-individual variations where possible. We considered four possible sources of variation: (1) acoustically-unrelated “baseline” neuronal activity, (2) neural activity differences due to spontaneous motor behavior, (3) neural activity differences related to acoustically-evoked motor behavior performance (i.e., SLC, LLC, NR), and (4) neural activity differences in sensation and processing underlying behavior selection, learning, and memory. We typically sought to reduce the first two sources of variation for most paradigms, and the 3rd or 4th depending on the research question at hand. Despite identical acoustic stimuli across conditions, modifying UV delivery parameters allowed us to capture and extract distinct regional activity patterns between experimental groups (Figures 1–4).

Reducing the total cumulative UV, such as from 45 s to 22.5 s (Figure 2), enhanced our ability to detect acoustically-evoked activity, and this should generally reduce the impact of acoustically-unrelated baseline and motor activities. We did not explore the lower limits of detection by CaMPARI2, though reducing from 25 s to 12.5 s total exposure still effectively detected our activity of interest (Figure 4), though this was accompanied by reduced fold change values. While we predicted that acoustically-unrelated activity would be randomly sampled across UV exposure windows, this may still vary by internal state differences between individuals such as motivation, attention, hunger, or stress (Johnson et al., 2020; Krishnan et al., 2025; Légaré et al., 2025).

Shortening each UV window, such as from 3 s to 1.5 s or 250 ms to 125 ms (Figures 1–4), reduced the likelihood of capturing spontaneous motor behavior in each window, since larval movements are performed in discrete discontinuous bouts (Marques et al., 2018). With acoustically-evoked escape behavior, sufficiently small windows should exclude spontaneous behavior almost entirely, since performing escape bouts precludes other motor bouts (Burgess and Granato, 2007b). PC windows as small as 125 ms were sufficient to permit meaningful comparison between ROIs for acoustically-evoked escape behavior (Figure 4) even without other compensation, compared to 250 ms windows (Figure 3). Furthermore, activity variation within ROIs among individuals within the –125 baseline condition (Figure 4B) were noticeably milder than in the –250 baseline condition (Figure 3D), consistent with reduced capture of spontaneous movement bouts. We speculate that even shorter photoconversion windows may also be effective, and perhaps even distinguish between distinct movement patterns within behavioral bouts. While the achievable resolution is constrained by the calcium binding and photoconversion kinetics of CaMPARI2, it is likely sufficient to distinguish between different movement periods and consolidation or decision-making phases of behaviors occurring across more extended time periods than rapid escape maneuvers examined here.

Increasing the number of UV-captured stimuli, such as from 15 to 100 (Figures 1–3), enhanced both the RGR of known acoustically-related ROIs captured and the magnitude of fold change of R/G over matched UV controls. Since CaMPARI2 integrates across captured epochs, increasing captured events enhances consistency when relevant behaviors aren’t performed 100% of the time (Figures 2B, 3B,C), and increasing the number of captured stimuli can offset lower signal from short UV capture duration.

Increasing the ISI between UV-captured stimuli, as we did from 30 s to 45 s during piloting (Figures 1A, 2A), increased the average SLC performance rate. In our “low habituation” condition overall SLC response rates significantly declined from stimulus 1–100 (Figure 5C). We anticipate that further increasing or altering the ISI would further reduce habituation to better distinguish different levels or periods of habituation learning (Wolman et al., 2011; Pantoja et al., 2016; Nelson et al., 2023). Since increasing numbers of UV-captured stimuli would also increase capture of “baseline” neural activity, it is important to balance this against potential other changes in behavioral states (Horstick et al., 2016).

Altering the timing of UV relative to stimuli of interest, such as shifting UV windows before or after acoustic stimuli while holding other variables constant (Figures 3, 4), is critical to establish a window that closely matches the circuit function period of interest. Acoustically-evoked escape behaviors initiate in a restricted and stereotyped latency range (0–15 ms for SLC, 15–60 ms for LLC) and bout duration range, facilitating our UV window selection (Burgess and Granato, 2007b; Zúñiga Mouret et al., 2024). We found short windows of UV exposure optimally captured neuronal activity underlying acoustically-evoked motor behavior that had occurred 0.5–1 s *prior to the UV onset*, consistent with electrically-evoked activity in culture and slice preparations (Moeyaert et al., 2018; Perez-Alvarez et al., 2020). The +750 ms condition showed similar levels of PC, but with greater consistency between replicates across the whole brain (Figure 3C) and an acoustically relevant ROI subset (Figure 3D). ROIs displaying the largest stimulus-evoked activity increases remained fairly consistent between conditions, with those most active in the +625 ms condition overlapping most substantially with those previously identified by 250-ms UV exposure conditions (Figures 3G, 4D). Taken together, our results point to a stimulus-to-UV delay time of 500–750 ms as optimal to capture rapid acoustically-evoked escape behaviors, though we predict that exploring a more expansive range of longer UV delay times may be informative for less vigorous or longer latency responses, or to capture evoked circuit activity changes operating at longer timescales such as sensorimotor adaptation, associative learning, or behavioral state changes (Aizenberg and Schuman, 2011; Andalman et al., 2019; Lovett-Barron, 2021; Markov et al., 2021).

Altering the context surrounding photoconversion periods, such as using different ISIs between captured acoustic stimuli, can allow differentiation between learning state related activity. Similarly, introducing additional acoustic stimuli that were *not* accompanied by UV prior to the UV-captured stimuli (Figures 5A, 6A) shifted the behavioral profile of larvae. Providing acoustic stimuli outside of the UV capture window for control individuals (Figures 3, 4) ensures that any larger time scale behavioral state changes occurring for fish should be accounted for to focus on acute behavior selection and performance. Notably, excluding capture of neural activity interleaved or immediately prior to stimuli is not possible with IEG-based approaches.

The CaMPARI2 system allowed us to directly compare stimulus-locked differential neural activity between larvae in naive and habituated behavioral states, comparing integrated neuronal activity states captured across 100 stimuli over 75 min vs. 100 stimuli over 10 min (Figure 5A). In contrast, IEG- and pERK-based methods aggregate neural activity over the entirety of their extended detection windows, where detection is instead determined by the response and decay time course of the marker used, precluding comparisons between conditions in which the same number of stimuli are distributed over markedly different timescales. This temporal restriction difference allows the CaMPARI2-based approach to resolve both sustained, brain-wide changes associated with habituation and acute, stimulus-evoked differences in neuronal responses, despite the large differences in ISI and total experimental duration.

### 3.2 Circuits modulating acoustically-evoked escape behavior and learning

Principal component analysis revealed that low- and high-habituated larvae form largely distinct clusters around PC1, which captured the majority of variance between the two stimulus delivery conditions (Figure 5E). In conjunction with consistent patterns of activity increases and decreases localized to particular ROIs (Figure 5D), this indicated that CaMPARI2 was able to resolve consistent, coordinated patterns of activity defining the two behavioral states.

To relate global separation observed in PCA to ROI-level habituation-driven activity, we examined the ROIs most strongly driving that separation, providing mechanistic insight into how whole-brain state differences emerge from region-level stimulus-evoked activity. Notably, several ROIs loading most strongly into the dominant principal component also ranked among the most strongly habituation-enhanced ROIs (Figures 5F, 5K). The convergence of these analyses indicates that behavioral state separation is driven by coordinated upregulation of particular ROIs, pointing toward specific circuit elements selectively engaged during habituation. The overlap between ROIs loading most strongly into the second principal component and habituation-depressed ROIs suggests that these particular regions contribute to orthogonal, inter-individual variability in stimulus responses rather than driving primary behavior state separation (Figures 5G,I).

The ROIs most strongly segregating larvae by behavioral state and displaying the highest habituation-enhanced activity belonged to either the preoptic area or the subpallium (Figures 5F, 6F), regions broadly connected to sensory adaptation and learning (Portavella et al., 2004; Lal et al., 2018; Nelson et al., 2023; Pandey et al., 2023; Palieri et al., 2024). The zebrafish preoptic area contributes to homeostatic navigation based on deviations from optimal water temperature, playing a vital role in behavioral modulation based on changing sensory context (Palieri et al., 2024). Though functionally heterogeneous, various regions of the subpallium have been implicated in fear and avoidance learning in teleost fish as well (Portavella et al., 2004; Lal et al., 2018; Pandey et al., 2023). In contrast to our results, habituation-deficient *pappaa* and *ap2s1* mutant larvae display consistent elevated subpallial and preoptic area activity over siblings (Nelson et al., 2023). Because these mutants show constant elevated pERK activity in these regions even without stimulation, this might preclude pERK-based detection of the temporally-specific recruitment of subpallial and preoptic area subpopulations we observed in wild type individuals through CaMPARI2. Therefore, more detailed cell- and time-resolved assessment of neurons promoting habituation within these regions is necessary to determine the direct contributions of these neurons to habituation learning.

In contrast to the consistent and coordinated activity patterns observed in our habituation experiment, we noted that MK-801-treated larvae exhibited markedly increased replicate-to-replicate variability (Figure 6D), indicating that CaMPARI2 captured the erosion of separation between habituated and non-habituated larvae following administration of MK-801. pERK-detection work has found that MK-801 dampens activity in the subpallium and habenula, which our findings replicated (Figures 6G,H). Genetic disruption of neurotransmission in the zebrafish dorsal habenula impairs the modulation of responses to conditioned aversive stimuli, an associative learning process (Agetsuma et al., 2010). Additionally, dorsolateral-habenula-ablated juvenile zebrafish show a deficit in their ability to integrate past and novel experiences in a memory extinction and reversal learning context, positioning the region as vital for continuous behavioral modulation (Palumbo et al., 2020). Left dorsal habenula function is required for recovery of larval swimming behavior following a fear-inducing stimulus and subsequent response (Duboué et al., 2017). The lateral habenula has also been implicated in the transition between active and passive behavioral coping in larvae, demonstrating progressive encoding of sensory experience (Andalman et al., 2019).

Together, these observations demonstrate the relevance of preoptic, subpallial, and habenular populations in habituation learning and highlight the dependence of differential activity interpretation on the temporal specificity of capture. Future cell-level characterization of these regions will reveal their precise functionality in habituation learning.

### 3.3 Opportunities and limitations for leveraging CaMPARI2 to understand behavioral circuitry in zebrafish

Our results demonstrate that brief windows of UV exposure optimally capture neuronal activity occurring 0.5–1 s prior to UV onset (Moeyaert et al., 2018; Perez-Alvarez et al., 2020). Both the UV-dependence and the delay inherent to this technique provide an opportunity for integration with closed-loop paradigms, in which behavioral responses influence the stimuli presented to an animal in real time (Jouary et al., 2016; Kawashima et al., 2016; Naumann et al., 2016; Vanwalleghem et al., 2018). Similarly, delivering photoconverting UV light contingent upon and temporally synchronized to the execution of particular behaviors offers an avenue for investigating specific behavioral stages of complex and temporally variable scenarios, which would be difficult to capture with predetermined, temporally-fixed stimulus and UV delivery paradigms. Using this tool to provide mechanistic insight on other pharmacological or genetic variants shifting sensorimotor gating sensitivity or selection bias between escape responses, where capturing temporally precise subsets of neural activity is essential (Burgess and Granato, 2007b; Wolman et al., 2015; Jain et al., 2018; Marsden et al., 2018). Combining zebrafish whole-brain CaMPARI2 studies with genetic variants linked to human neuropsychiatric conditions would facilitate mechanistic neurobiological understanding, though assessing neurodevelopmental structural differences will be critical to appropriately interface with standardized brain atlases (Gupta et al., 2018; Thyme et al., 2019; Marquez-Legorreta et al., 2022; Mendes et al., 2023; Capps et al., 2025). While the temporal specificity and stereotyped nature of acoustically-evoked behavior make CaMPARI2 an effective tool to dissect the underlying neural activity, this pipeline could be implemented to investigate behaviors engaging other sensory modalities where restraint can compromise behavioral features, such as hunting or social behaviors (Tunbak et al., 2020; Zhao et al., 2025).

At the same time, the dependence of CaMPARI2 activity capture on UV light introduces important considerations. Zebrafish larvae exhibit high spectral sensitivity to UV light and display intensity-dependent avoidance behavior, raising the possibility that UV exposure itself could disrupt visually guided behaviors, influence multisensory integration, or act as an unintended associative learning cue (Nava et al., 2011; Guggiana-Nilo and Engert, 2016). Indeed, during our piloting experiments we noted altered responsiveness and habituation rates when UV flashes were incorporated into the stimulus paradigm at different points relative to acoustic stimuli, consistent with light-evoked sensitization of Mauthner-dependent escapes (Mu et al., 2012). We also observed that sufficiently short UV pulses fail to evoke light flash induced responses during piloting, suggesting UV photoconversion doesn’t necessarily preclude visually-guided behavior analysis through CaMPARI2, consistent with its recent use capturing mouse visually-guided behaviors (Burgess and Granato, 2007a; Das et al., 2023a). However, pERK and *c-fos* based methods may complement or be better suited than CaMPARI2 in UV-sensitive behavioral contexts by completely sidestepping the need for UV, or when detecting neural activity patterns over variably extended and/or poorly time-locked periods since these IEG-based methods integrate activity over minutes to hours without introducing new stimuli (Barykina et al., 2022). Regardless, our behavioral observations underscore the necessity of incorporating UV flashes into reference conditions and behavioral assay optimization.

Combining CaMPARI2 with complementary experimental approaches offers the opportunity to link whole-brain activity patterns to subcellular activity dynamics and molecular determinants of circuit function. Incorporation of pre- and post-synaptically targeted CaMPARI2 variants like SynTagMA would permit activity capture at the synaptic level, enabling finer dissection of particularly distributed circuits spanning large brain areas (Perez-Alvarez et al., 2020). Recent work in both mice and larval zebrafish has also combined CaMPARI-based activity capture with fluorescence-activated cell sorting and subsequent transcriptomic profiling, allowing populations recruited during particular behavioral states to be characterized and linking circuit dynamics to gene expression programs (O’Toole et al., 2023; Matsuda et al., 2025). Both CaMPARI2 whole-brain activity maps and transcriptomic datasets derived from CaMPARI2-labeled neurons lend themselves to a variety of computational analyses. Applied to whole-brain activity, multivariate, clustering, and network-based approaches can uncover coordinated patterns of circuit engagement and behavioral-state-dependent differences in whole-brain activity, as demonstrated by distinct clustering of habituation states in principal component space (Constantin et al., 2020; Marquez-Legorreta et al., 2022; Légaré et al., 2025). In contrast, the absence of comparable clustering of pharmacologically-induced behavior states highlights how such analyses can reveal more heterogenous, globally dysregulated activity patterns, consistent with the variable and widespread effects of NMDA receptor antagonism (Moghaddam et al., 1997; Homayoun and Moghaddam, 2007). Additionally, different experimental perturbations require tailored computational frameworks to faithfully assess their impact on neural activity (Cunningham and Yu, 2014). Altogether, expanding the zebrafish toolkit for whole-brain circuit activity analyses using CaMPARI2 effectively complements existing methods and expands the accessibility of large-scale behavioral circuit dissection beyond highly specialized real-time volumetric imaging equipment.

## 4 Materials and methods

### 4.1 Zebrafish maintenance and husbandry

Zebrafish maintenance, husbandry, and raising of larvae was performed in accordance with standard published guidelines. All experimental protocols were approved by the Haverford College IACUC. Zebrafish were maintained on a 14 h light/10 h dark cycle. Embryos and larvae were raised in 1× E3 (5 mM NaCl, 0.17 mM KCl, 0.33 mM CaCl_2_, 0.33 mM MgSO_4_) at 29°C. Larvae used for whole-brain confocal imaging were raised in 200 μM phenylthiourea (PTU, Sigma, P-7629) in 1× E3 beginning at 24 h post-fertilization (hpf) to inhibit melanophore development. Embryos were treated with 20 μg/mL pronase (Sigma, 10165921001) between 20 and 28 hpf to facilitate synchronous dechorionation. Pronase was removed and replaced with fresh PTU/E3 after 24 h of exposure. Prior to imaging, larvae were screened for central nervous system CaMPARI2 fluorescence and developmental abnormalities. Larvae between 5 and 7 dpf were used for all imaging and behavioral experiments. As there are currently no consistent known genetic markers for sex in zebrafish and sexual differentiation does not occur until 25–60 dpf, all larvae were tested without regards to sex. Larvae were humanely euthanized via rapid chilling or tricaine overanesthesia following experiments in accordance with AVMA guidelines. All CaMPARI2 imaging experiments presented in this manuscript used the *Tg(elavl3:CaMPARI2)jf92* transgenic line, referred to as *elavl3:CaMPARI2* (ZDB-ALT-180410-5) (Moeyaert et al., 2018), though some original pilot experiments were performed using the earlier *Tg[elavl3:CaMPARI(W391F* + *V398L)]jf9* line (ZDB-ALT-150403-1) (Fosque et al., 2015). Transgenes were maintained in the Tüpfel long fin (TLF) background (ZDB-GENO-990623-2) carrying a *mitfa* mutation (ZDB-ALT-060913-2) to reduce skin pigmentation for enhanced imaging quality. For all behavioral assays here, the non-transgenic TLF strain was used with normal pigmentation to facilitate automated behavioral tracking.

### 4.2 CaMPARI2 photoconversion and acoustic behavior assays

For all photoconversion and subsequent brain imaging, 5–7 dpf *elavl3:CaMPARI2* larvae displaying bright green central nervous system fluorescence were selected to ensure sufficient CaMPARI2 expression. Four larvae were placed together into one 9 mm diameter circular well of a 36-well custom plexiglass arena attached to a vibrational exciter (Hottinger Brüel & Kjær, 4810) delivering non-directional vibroacoustic stimuli. Acoustic stimuli were administered using DAQTimer software (Burgess and Granato, 2007a) at a frequency of 1,000 Hz, intensity of 23.5 dB, and duration of 2 ms, as previously described (Zúñiga Mouret et al., 2024), and at the intervals specified in the assay diagrams presented (Figures 1A, 2A, 3A, 4A, 5A, 6A). UV light was delivered via a lightguide-coupled LED light source (UHP-T-405-DI, Prizmatix) with a spectral maximum of 405 nm and maximum output of 5 W, and was also controlled via DAQTimer to precisely synchronize UV illumination with acoustic stimuli and/or high-speed camera recordings. For photoconversion experiments, the UV light source was placed directly above the well containing the larvae, approximately 2 cm from the surface of the water to provide consistent illumination. 405 nm irradiance of 1,103 mW/cm^2^ was measured at the well under these conditions, using a LIGHTmetric ONE spectral light meter (Gigahertz-Optik).

Since the photoconversion UV light source occluded larvae from overhead video recording, in our representative behavioral analyses, the UV light was presented obliquely from above approximately 20 cm from the surface such that UV light would fully illuminate all wells containing fish. A high-speed camera (TS4, Fastec) fitted with a Sigma 50 mm f/2.8 EX DG Macro Autofocus lens (B&H Photo) and 720 nm wavelength filter (Opteka) filter was used to record larval behavior at 1,000 fps in 1,000 ms bursts (Figure 2B) or 280 ms bursts (Figures 3–6), triggered 30 ms prior to each acoustic stimulus. An infrared array (CM-IR200-850, CMVision) provided illumination for the camera below the white plexiglass diffuser base of the testing arena to allow recordings with and without 405 nm illumination, as previously described (Zúñiga Mouret et al., 2024). For representative behavioral analyses, each 9 mm well either contained four wild type TLF larvae (Figure 2B) or single wild type TLF larvae (Figures 3–6) raised without PTU, unlike larvae destined for photoconversion, to preserve normal body pigment and permit automated behavioral tracking via the FLOTE software package (Burgess and Granato, 2007a). This software classifies behavioral bouts based on a combination of kinematic features, and we used latency thresholds of 1–15 ms (SLC) and 16–80 ms (LLC) post-acoustic stimuli when distinguishing escape behaviors from “Other” behavioral bouts, which include swim bouts and lower angular velocity “routine turns.” All behavioral experiments were carried out at 6 or 7 dpf, and compared between matched sibling larvae.

Since individual larvae could not be continuously tracked through the entire experiment of Figure 2B, population-level behavior was analyzed to determine the percent of fish performing SLC, LLC, Other, and No Response (NR) across the entire population during each 1,000 ms block recorded (UV illuminated, with or without acoustic stimuli). Each point corresponded to a single UV exposure, with 15 exposures per condition at 45 s ISI. The remaining behaviorally-tracked experiments (Figures 3, 5, 6) were performed with single fish in isolated wells, so that behavioral profile differences between individuals could be assessed across the entire experiment. Thus, each individual was scored for the percentage of stimuli in which they performed each behavioral bout class across 100 UV-illuminated stimuli (Figures 3B, 3C, 5B, 6B). Behavioral time courses evoking habituation only report the overall percent of fish performing an SLC behavior at each stimulus (Figures 5C, 6C).

### 4.3 Pharmacological treatment of larvae

NMDAR glutamate neurotransmission was acutely disrupted as previously described (Wolman et al., 2011; Nelson et al., 2023). A 100 mM stock of MK-801 was prepared by dissolving powdered (+)-MK-801 hydrogen maleate (Sigma Aldrich, M107) in 100% DMSO. Prior to photoconversion, five 5–7 dpf larvae were placed into one 21.9 × 17.5 mm (diameter × height) circular well of a standard 12-well plate containing 2 mL of 1× E3. Thirty minutes prior to the onset of UV and stimulus delivery, 10 μL of either 100% DMSO or 100 mM MK-801 was added to the wells, producing final treatment concentrations of 0.5% DMSO and 500 μM MK-801.

### 4.4 Whole-brain imaging of larvae

Following photoconversion and stimulation, larvae were anesthetized with buffered 0.01–0.015% tricaine (Syndel) pH 7 in 1× E3 and mounted dorsal-down in 80 μL 1.5% low-melting point agarose (Sigma, A9414) on 35 mm coverslip bottom dishes (Mattek) and overlaid with diluted tricaine/E3. Larvae were mounted with the forebrain and optic tectum flush against the slipcover with minimal lateral tilt. Whole-brain, multichannel z-stacks were collected on a Leica Stellaris 5 line-scanning confocal microscope using a 20× water immersion objective. Unconverted CaMPARI2 fluorescence was collected in the green channel with 488 nm excitation at 40.08% intensity and a detection range of 495–554 nm with a gain of 33.0% (Moeyaert et al., 2018). Photoconverted CaMPARI2 fluorescence was collected in the red channel with 561 nm excitation at 90.08% intensity and a detection range 566 and 700 nm with a gain of 70.0% (Moeyaert et al., 2018). Two overlapping 512 × 512 z-stacks were collected for each brain (1 μm z-step size, bidirectional scanning, collecting green then red channels sequentially for each 512 × 512 stack) and automatically stitched together through the Leica LAS X software. Z-stack endpoints were set just below and above the most dorsal and ventral portions of the brain, respectively, to capture the entire brain at a voxel resolution of 1.14 μm × 1.14 μm × 1.0 μm. For representative brain image presentation (Figures 1C–H and Supplementary Figure 1), image stacks were captured at identical settings, and the photoconverted channel was isolated and switched to the Fire LUT before z-projection in Fiji/ImageJ (Schindelin et al., 2012).

### 4.5 ROI-based fluorescence quantification

Pre-processing, registration, and fluorescence quantification of whole-brain images was based on the Multivariate Analysis of Variegated Expression in Neurons protocol (Shoenhard and Granato, 2023). Merged multichannel z-stacks in LIF (Leica image file) format were opened in Fiji/ImageJ (Schindelin et al., 2012) and reoriented such that the forebrain pointed upwards. The red and green channels were separated and stack-sorted such that images were ordered from the ventral to dorsal direction, and saved individually as NRRD files with suffixes denoting each channel. Files were then registered to the Z-brain atlas ZBB_jf9_huC-CaMPARI reference brain with the Computational Morphometry Toolkit (CMTK) Fiji plug-in for Mac OS (Randlett et al., 2015; Shoenhard and Granato, 2023). Registered NRRD files were downsampled, smoothed, and converted to TIFF files using the PrepareStacksForMAPMapping.ijm Fiji macro (Randlett et al., 2015). Mean red and green voxel fluorescence was quantified in each of 293 neuroanatomical regions (as defined by the Z-brain atlas) with the QuantifySignalMultipleBrains.m MATLAB script, which uses the MaskDatabaseDownsampled.mat and AnatomyLabelDatabase.hdf5 reference files (Randlett et al., 2015). All scripts were unmodified.

Regional fluorescence quantification returns a CSV file of red and green fluorescence in each ROI for each larva. All subsequent data processing was performed in Python (v3.14) with pandas and NumPy. Red fluorescence was normalized to green fluorescence by calculating a red-to-green ratio (RGR) for each ROI and larva. For each ROI, the median RGR was calculated across control replicates to capture activity unrelated to the behavior of interest as a reference. RGR values from experimental and control replicates were divided by the corresponding median value of the control population to calculate fold activity changes. For all downstream analyses and visualizations, ROIs and larvae with > 10% of fluorescence values missing were excluded. We excluded the following ROIs from all of our analyses presented here because their superficial positions prevented consistent or reliable fluorescence readings across warped brains: facial glossopharyngeal ganglion, posterior and anterior lateral line ganglia, and lateral line neuromasts D1, D2, N, O1, OC1, SO1, SO2, and SO3. We also excluded the ROI containing the Mauthner cell bodies from presented data, as these particular neurons do not appear to reliably express CaMPARI2 in the *jf92* transgenic line used. Thus up to 281 distinct ROIs were analyzed for each fish. The web-based FishExplorer interface^1^ was used to generate 3D diagrams of ROIs in the context of the full larval brain (Vohra et al., 2025).

### 4.6 Data visualization and statistical analyses

Behavioral frequencies (Figures 2B, 5B, 6B) were compared via multiple Mann-Whitney tests with corrected *p*-value threshold of α = 0.05, Holm-Šídák method, conducted using Prism 10.6 (GraphPad). All ROI data visualization was conducted in Python (v3.14) using Matplotlib, seaborn, and scikit-learn. To generate heat maps, fold changes were log_2_ transformed to render activity increases and decreases symmetrical and enable comparison between ROIs. We thresholded log_2_(fold change) values at –1.5 and 1.5 for consistent visualization purposes in all heat maps. Heat map columns within each experimental condition are sorted by the median log_2_(FC) value of each brain, from smallest to largest.

PCA was performed on the ROI-level RGR matrix after mean-centering each ROI across larvae. Individual larvae were treated as observations and ROIs as features, with each larva represented by a vector of RGR values across ROIs. Values missing after data filtering were imputed using the mean RGR value for that ROI across larvae. PCA was performed on the full larva-by-ROI matrix and the first two principal components were computed and visualized. ROIs contributing most strongly to each component were identified from the corresponding loading vectors.

## Data Availability

The raw data supporting the conclusions of this article will be made available by the authors, without undue reservation.

## Footnotes

1
https://fishexplorer.zib.de/sandbox/
