# Supplementary figures and images for: CaMPARI2 enables stimulus-locked whole-brain activity mapping at cellular resolution in unrestrained larval zebrafish
Source: Front Mol Neurosci. 2026 Apr 14;19:1772915. doi: 10.3389/fnmol.2026.1772915 (PMC13121368; doi:10.3389/fnmol.2026.1772915)

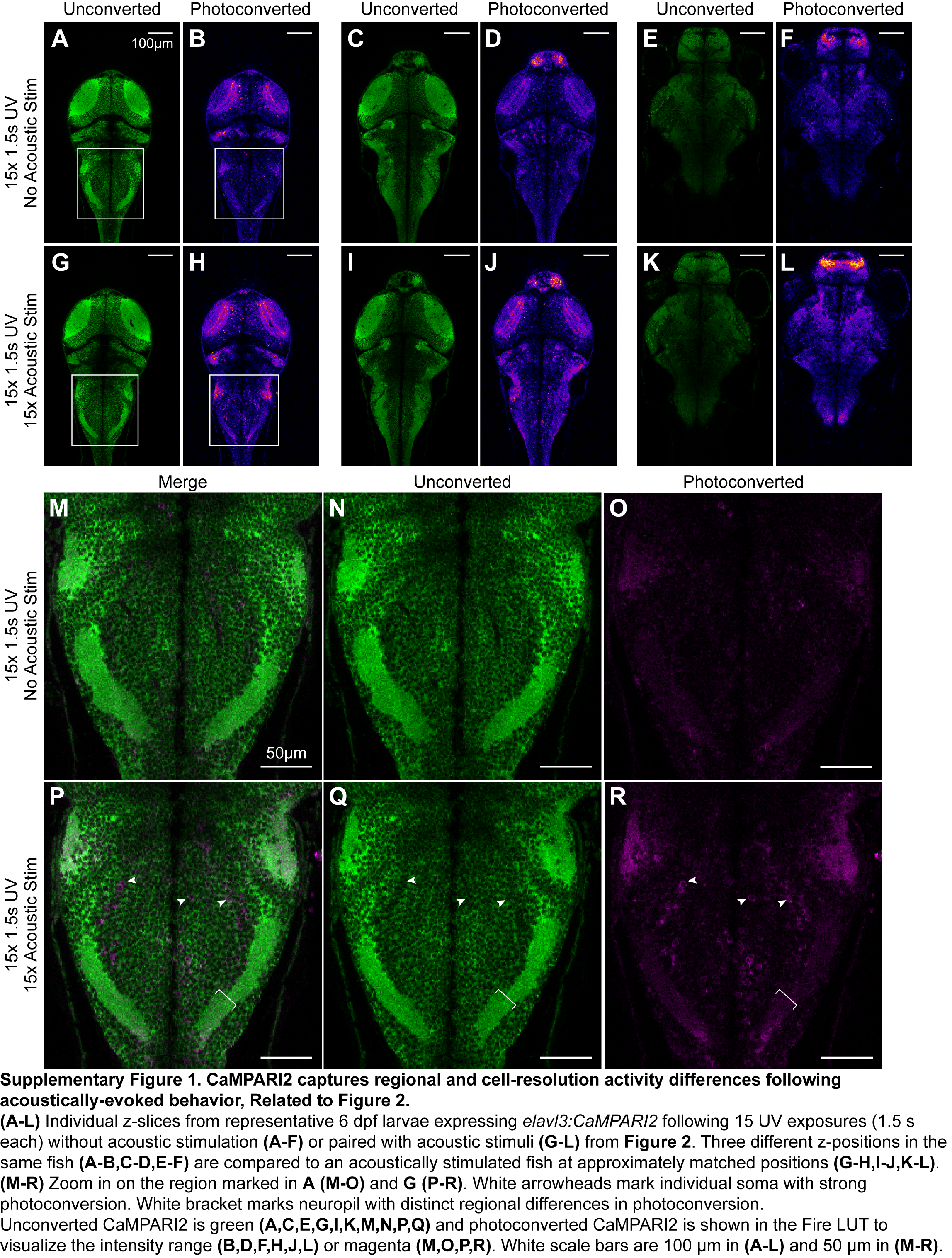

Supplement: Supplementary file 1 [file Image_1.tif]
